# Incorporating published univariable associations in diagnostic and prognostic modeling

**DOI:** 10.1186/1471-2288-12-121

**Published:** 2012-08-10

**Authors:** Thomas P A Debray, Hendrik Koffijberg, Difei Lu, Yvonne Vergouwe, Ewout W Steyerberg, Karel G M Moons

**Affiliations:** 1 Julius Center for Health Sciences and Primary Care, University Medical Center Utrecht, Utrecht, The Netherlands; 2 Center for Medical Decision Sciences, Department of Public Health, Erasmus Medical Center, Rotterdam, The Netherlands

## Abstract

**Background:**

Diagnostic and prognostic literature is overwhelmed with studies reporting univariable predictor-outcome associations. Currently, methods to incorporate such information in the construction of a prediction model are underdeveloped and unfamiliar to many researchers.

**Methods:**

This article aims to improve upon an adaptation method originally proposed by Greenland (1987) and Steyerberg (2000) to incorporate previously published univariable associations in the construction of a novel prediction model. The proposed method improves upon the variance estimation component by reconfiguring the adaptation process in established theory and making it more robust. Different variants of the proposed method were tested in a simulation study, where performance was measured by comparing estimated associations with their predefined values according to the Mean Squared Error and coverage of the 90% confidence intervals.

**Results:**

Results demonstrate that performance of estimated multivariable associations considerably improves for small datasets where external evidence is included. Although the error of estimated associations decreases with increasing amount of individual participant data, it does not disappear completely, even in very large datasets.

**Conclusions:**

The proposed method to aggregate previously published univariable associations with individual participant data in the construction of a novel prediction models outperforms established approaches and is especially worthwhile when relatively limited individual participant data are available.

## Background

Recent medical literature has shown an increasing interest in clinical prediction models obtained from cross-sectional studies (diagnostic models) as well as case-control, cohort and randomized controlled data (prognostic models) [1-5]. Such models combine multiple predictors or markers that are independently associated with the presence (in case of diagnosis) or future occurrence (in case of prognosis) of a particular outcome. Typically, logistic regression is used to model these binary outcomes. Alternatively, Cox proportional hazards regression may be applied to account for the time-to-event.

The development of a novel prediction model requires a dataset with a sufficient amount of participants to obtain accurate associations and to make reliable predictions. Also, larger numbers of participants increase the statistical power when selecting predictive subject characteristics to be included in predictive models. Although numerous prediction models are constructed from a single dataset, it is possible to increase the amount of evidence available by incorporating information from the literature.

The availability of individual participant data (IPD) is commonly recommended as gold standard for combining existing information with newly collected data [6,7]. However, this situation is often unfeasible due to practical constraints [8,9], for instance when studies were conducted several years ago. Fortunately, numerous papers contain baseline population characteristics from which univariable predictor-outcome associations can be derived. Consequently, these associations represent an appealing source of evidence when developing a novel prediction model [5,10-17].

Greenland and Steyerberg have recently proposed adaptation methods to incorporate previously published univariable predictor-outcome associations as prior evidence in a regression analysis [18,19]. These methods combine the result of a univariable meta-analysis with the results of a univariable and multivariable logistic regression analysis on the IPD. Although these quantitative approaches may considerably improve the quality of a model’s regression coefficients and its resulting performance, they are not yet frequently used in practice [20,21].

Here we present an improved alternative to the methods proposed by Greenland and Steyerberg that aims to further increase the accuracy and precision of the multivariable associations estimated using external evidence. This method improves upon the variance estimation component by reconfiguring the adaptation process in established theory and making it more robust. We present two variants of our method and test their performance in a simulation study. We illustrate the proposed methods’ application in a clinical example involving the prediction of peri-operative mortality after elective abdominal aortic aneurysm surgery [22].

## Methods

This method is intended to address the specific situation where IPD have been collected to evaluate the effect of a number of predictors on a dichotomous outcome using logistic regression analysis. Here, univariable and multivariable associations (logistic regression coefficients) are estimated and denoted as *β*_u_ and *β*_m_. Particularly, two sources of associations are assumed to be available, namely the IPD of the study at hand ( I ) and aggregated data from the literature ( L ). The univariable and multivariable associations estimated in the derivation data are denoted as ${\hat{\beta }}_{u|\text{I}}$ and ${\hat{\beta }}_{m|\text{I}}$. For the literature, only univariable associations are available ( ${\hat{\beta }}_{u|\text{L}}$ ). It is assumed that the study at hand and the studies forming the literature are both random samples from a common underlying patient population.

Previously, Greenland proposed a method to incorporate univariable associations reported in the literature when developing a novel multivariable prediction model from newly collected data [18]. This method attempts to approximate a situation where the individual participant data from all the previously published datasets was available for all the candidate covariates. It uses the calculated change from univariable to multivariable association in the newly collected data and uses this difference to estimate the multivariable association that would have been reported in the previous literature using the IPD from the previous studies:

(1) $${\hat{\beta }}_{m|\text{L}}={\hat{\beta }}_{u|\text{L}}+\left({\hat{\beta }}_{m|\text{I}}-{\hat{\beta }}_{u|\text{I}}\right)$$

The proposed estimate for the variance of ${\hat{\beta }}_{m|\text{L}}$ is given as follows [18,23].

(2) $$\hat{\mathrm{Var}}\left({\hat{\beta }}_{m|\text{L}}\right)=\hat{\mathrm{Var}}\left({\hat{\beta }}_{u|\text{L}}\right)+\left[\hat{\mathrm{Var}}\left({\hat{\beta }}_{m|\text{I}}\right)-\hat{\mathrm{Var}}\left({\hat{\beta }}_{u|\text{I}}\right)\right]$$

Here, ${\hat{\beta }}_{u|\text{L}}$ can be obtained through a meta-analysis involving fixed or random effects, and ${\hat{\beta }}_{m|\text{L}}$ is the (asymptotically) unbiased estimate of the multivariable association ${\hat{\beta }}_{m}$. Subsequently, Steyerberg *et al.* extended this method by defining a weight *c* to reflect inconsistencies and variability in previous research [19]:

(3) $${\hat{\beta }}_{m|\text{L}}={\hat{\beta }}_{m|\text{I}}+c\left({\hat{\beta }}_{u|\text{L}}-{\hat{\beta }}_{u|\text{I}}\right)$$

Previous simulations have however shown that the original unweighted method (*c* = 1 in expression 3) has a similar performance.

### Concerns and proposed solutions

Although aforementioned formulas are relatively simple to apply, the calculation of $\hat{\mathrm{Var}}({\hat{\beta }}_{m|\text{L}})$ in expression 2 clearly contrasts with the theoretical variance component:

(4) $$\begin{array}{lll} \;\mathrm{Var}\left({\hat{\beta }}_{m|\text{L}}\right) & =\mathrm{Var}\left({\hat{\beta }}_{u|\text{L}}\right)+\mathrm{Var}\left({\hat{\beta }}_{m|\text{I}}\right)+\mathrm{Var}\left({\hat{\beta }}_{u|\text{I}}\right)\; & \;\\ & \;+2\;\mathrm{Cov}\left({\hat{\beta }}_{u|\text{L}},{\hat{\beta }}_{m|\text{I}}\right)-2\;\mathrm{Cov}\left({\hat{\beta }}_{m|\text{I}},{\hat{\beta }}_{u|\text{I}}\right)\; & \;\\ & \;-2\;\mathrm{Cov}\left({\hat{\beta }}_{u|\text{L}},{\hat{\beta }}_{u|\text{I}}\right)\; \end{array}$$

Although it is possible to assume that estimated associations from the literature and IPD at hand are independent, i.e. $\mathrm{Cov}({\hat{\beta }}_{u|\text{L}},{\hat{\beta }}_{m|\text{I}})=\mathrm{Cov}({\hat{\beta }}_{u|\text{L}},{\hat{\beta }}_{u|\text{I}})=0$, the remaining assumption that $\mathrm{Cov}({\hat{\beta }}_{m|\text{I}},{\hat{\beta }}_{u|\text{I}})=\mathrm{Var}({\hat{\beta }}_{u|\text{I}})$ seems unrealistic. Particularly, this assumption requires that the univariable and multivariable association in the IPD at hand are strongly correlated and neglects $\mathrm{Var}({\hat{\beta }}_{m|\text{I}})$, as $\mathrm{Cov}({\hat{\beta }}_{m|\text{I}},{\hat{\beta }}_{u|\text{I}})=\rho ({\hat{\beta }}_{m|\text{I}},{\hat{\beta }}_{u|\text{I}})\;\mathrm{Var}({\hat{\beta }}_{m|\text{I}})\;\mathrm{Var}({\hat{\beta }}_{u|\text{I}})$. Consequently, expression 2 may yield biased variance estimates of adapted multivariable associations. Although it is even possible that $\hat{\mathrm{Var}}({\hat{\beta }}_{m|\text{L}})$ becomes negative when $\hat{\mathrm{Var}}({\hat{\beta }}_{m|\text{I}})<\hat{\mathrm{Var}}({\hat{\beta }}_{u|\text{I}})$, this is unlikely to happen because adjustment of logistic regression coefficients is expected to result in a loss of precision [24].

In order to obtain asymptotically unbiased estimates for $\mathrm{Var}({\hat{\beta }}_{m|\text{L}})$, we incorporate the distribution of estimated associations. A pragmatic parametric family for the distribution of associations is the normal distribution, where we assume that ${\hat{\beta }}_{u|\text{I}}∼N(\mu_{u|\text{I}},\sigma_{u|\text{I}}^{2})$, ${\hat{\beta }}_{m|\text{I}}∼N(\mu_{m|\text{I}},\sigma_{m|\text{I}}^{2})$ and ${\hat{\beta }}_{u|\text{L}}∼N(\mu_{u|\text{L}},\sigma_{u|\text{L}}^{2})$. Then, the adaptation from univariable to multivariable association, i.e. ${\hat{\beta }}_{m|\text{I}}-{\hat{\beta }}_{u|\text{I}}$ in expression 1, is also normally distributed. The distribution of this adaptation is further denoted as $N\left(\mu_{\delta },\sigma_{\delta }^{2}\right)$ , such that ${\hat{\beta }}_{m|\text{L}}$ can be estimated by:

(5) $${\hat{\mu }}_{u|\text{L}}+{\hat{\mu }}_{\delta }$$

with a standard error estimate of

(6) $$\sqrt{{\hat{\sigma }}_{u|\text{L}}^{2}+{\hat{\sigma }}_{\delta }^{2}}$$

The probabilistic adaptation from univariable to multivariable association $N(\mu_{\delta },\sigma_{\delta }^{2})$ can be estimated from the IPD at hand using bootstrap sampling [25]. This procedure applies repeated sampling with replacement of subjects from the derivation dataset. Hence, it allows generating numerous datasets (bootstrap samples) where the adaptation can be estimated. Unfortunately, the bootstrap procedure may become unstable when the effective sample size is small, and yield regression coefficients with extreme values [26-28]. This, in turn, may strongly affect the quality of estimated adaptations and result in poor estimates of *β*_m|*L*_. For this reason, we propose to shrink the adaptation by implementing a Bayesian prior for the univariable and multivariable associations of the IPD at hand. Recently, Gelman *et al.* proposed a weakly default prior distribution that is based on the Cauchy distribution and assumes a probability of 70.48% for associations between -5 and 5. This distribution is less conservative than the uniform prior distribution (which assumes higher probabilities for extreme associations), and yields estimates that make more sense and have predictive performance better than maximum likelihood estimates [29]. The weakly informative prior distribution for generalized linear modeling was recently implemented in R, and is available in the package *arm*.

Finally, the summary of univariable associations from the literature $N(\mu_{u|\text{L}},\sigma_{u|\text{L}}^{2})$ is originally estimated by applying a fixed effects meta-analysis [30,31]. Because this estimate may be unstable when few studies are available, Steyerberg *et al.* proposed using the univariable associations from the literature (published as ${\hat{\beta }}_{u|\text{L}}$ ) and the IPD at hand (estimated as ${\hat{\beta }}_{u|\text{I}}$) [19]. When the homogeneity assumptions made by the adaptation method are violated, it is possible to assume random effects to further improve the robustness of estimated associations.

Given aforementioned concerns, we propose two variants (Table 1) of the adaptation method which we further denote as the *Improved Adaptation Method*. The first variant (*no prior*) decreases the bias of $\hat{\mathrm{Var}}({\hat{\beta }}_{m|\text{L}})$ by effectively removing the unrealistic assumptions about the covariance between univariable and multivariable associations in the IPD at hand. This variant also attempts to reduce the impact of heterogeneity by allowing random effects in the pooling of literature associations. The second variant (*weakly informative prior*) aims to further improve the quality of estimated multivariable associations by implementing a weakly informative prior distribution for estimating the univariable and multivariable associations in the IPD at hand. For this purpose, its logistic regression analyses use independent Cauchy distributions on all regression coefficients, each centered at 0 and with scale parameter 10 for the constant term and 2.5 for all other coefficients. In this manner, estimates for the adaptation from univariable to multivariable association become more robust.

**Table 1:** Overview of approaches

		**No meta-analysis**	**Greenland/Steyerberg**	**Improved adaptation method**	
			**adaptation method**	**Variant 1**	**Variant 2**
Step 1	Estimate associations in IPD				
	Implemented	Yes	Yes	Yes	Yes
	Association type	m	u+m	u+m	u+m
	Prior distribution	none	none	none	weakly informative
Step 2	Summarize univariable associations				
	Implemented	No	Yes	Yes	Yes
	Source	-	I+L	I+L	I+L
	Pooling Method	-	random effects	random effects	random effects
Step 3	Estimate adaptation from univariable to multivariable association				
	Implemented	No	Yes	Yes	Yes
	Assumptions	-	(1)+(2)	(1)	(1)
	Estimation procedure	-	analytic	bootstrap	bootstrap
	Prior distributions	-	none	none	weakly informative
Step 4	Apply adaptation to summary estimate from the literature and estimate *β*_m|L_				
	Implemented	No	Yes	Yes	Yes

This overview illustrates the characteristics of the approaches discussed and used in the simulation study. In the first step, univariable (u) and multivariable (m) associations are estimated in the IPD. In the second step, the univariable associations from the literature (L) and data at hand (I) are summarized. Afterwards, the adaptation from univariable to multivariable association is estimated in step 3. The assumptions about the variance component here are as follows: (1) estimated associations in the individual participant data (IPD) are independent from estimated associations in the literature, and (2) $\text{Cov}({\hat{\beta }}_{\text{m}|\text{I}},{\hat{\beta }}_{\text{u}|\text{I}})=\text{Var}({\hat{\beta }}_{\text{u}|\text{I}})$. Finally, step 4 estimates a multivariable association by applying the adaptation to the univariable summary estimate from the literature.

## Simulation study

**Figure 1:**
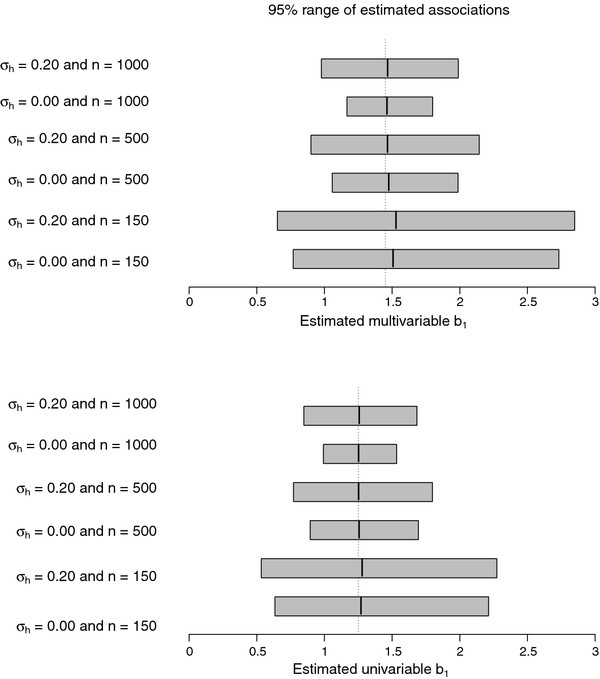
**Comparison of estimated associations.** Graphic presentation of multivariable (with true value 1.45) and corresponding univariable (with true value 1.25) associations estimated in an IPD of size *n*. This dataset is generated according to $x_{1},x_{2}∼N(0,1)$ with Pr(*y* = 1) = logit^−1^(−3.43 + b_1_*x*_1_ + 1.18*x*_2_) and ${\text{b}}_{1}∼N(1.45,\sigma_{\text{h}}^{2})$. Each interval is based on 10 000 repetitions.

We performed a simulation study to assess the quality of estimated multivariable associations. Hereto, we considered the situation in which IPD and literature data are described by two predictors and a dichotomous outcome. Arbitrary values were predefined for the independent association between these predictors and their respective outcome, with b_0_ = −3.43, b_1_ = 1.45 and b_2_ = 1.18 (where we chose $x_{1},x_{2}∼N\left(0,1\right)$ and $\rho \left(x_{1},x_{2}\right)=0$, i.e. *x*_1_ and *x*_2_ are not correlated) which we further refer to as the reference model. The outcome *y* for each subject *i* = 1, … , *N* is generated as follows, and corresponds to an average incidence of 9%.

$$\begin{array}{ll} y=\left\{\begin{array}{c} 1,\;\;\text{if}\;u\;<{\mathrm{logit}}^{-1}(-3.43+1.45\;x_{1}+1.18\;x_{2})\\ 0,\;\;\text{if}\;u\;\geq {\mathrm{logit}}^{-1}(-3.43+1.45\;x_{1}+1.18\;x_{2}) \end{array}\right) & \; \end{array}$$

where u∼U(0,1). We applied aforementioned methods (Table 1) to update only the multivariable association of the first predictor b_1_. In each scenario, data for four literature studies as well as an IPD are generated with different degrees of comparability. For this purpose, we used the reference model (fixed effects) to generate the IPD and source datasets of the univariable associations from the literature. We investigated the impact of sample size by evaluating different choices for *N*_I_ (100, 200, 500 and 1000) and *N*_L_ (500 and 2000). Note that *N*_I_ = 100 violates the rule of thumb that logistic models should be used with a minimum of 10 outcome events per predictor variable [28]. We also evaluated the performance for the scenario in which the key assumption of study exchangeability is violated. Hereto, we introduced random variation in b_1_ of the reference model when generating data for the literature studies:

$$\begin{array}{ll} y=\left\{\begin{array}{c} 1,\;\;\text{if}\;u\;<{\mathrm{logit}}^{-1}(-3.43+{\left(b_{1|L}\right)}_{j}\;x_{1}+1.18\;x_{2})\\ 0,\;\;\text{if}\;u\;\geq {\mathrm{logit}}^{-1}(-3.43+{\left(b_{1|L}\right)}_{j}\;x_{1}+1.18\;x_{2}) \end{array}\right) & \; \end{array}$$

where u∼U(0,1) and ${\left(b_{1|L}\right)}_{j}∼N\left(1.45,\sigma_{h}^{2}\right)$ with *j* = 1, … , 4. Consequently, differences in multivariable associations from the literature appear due to sampling variance and heterogeneity across study populations originated from one source of variability (e.g. due to a focus of studies on primary versus secondary care, younger versus older patients etc). Multivariable associations from the IPD at hand remain homogeneous with the study population (b_1|I_ = 1.45). The scenarios are illustrated in Figure 1, which also demonstrates that the sampling process substantially affects the bias and variance of the univariable and multivariable associations.

Finally, the updated multivariable association ${\hat{\beta }}_{1}$ obtained with each method is compared with the predefined association b_1_ from the reference model. We evaluate the frequentist properties of the estimated associations in terms of the percentage bias (PB) and the Mean Squared Error (MSE) [32], where

(7) $$\mathrm{PB}\left({\hat{\beta }}_{1}\right)=\frac{{\bar{\hat{\beta }}}_{1}-b_{1}}{b_{1}}\times 100\%$$

and

(8) $$\mathrm{MSE}\left({\hat{\beta }}_{1}\right)={\left({\bar{\hat{\beta }}}_{1}-b_{1}\right)}^{2}+{\left(\hat{\mathrm{SE}}\left({\hat{\beta }}_{1}\right)\right)}^{2}$$

In addition, we calculate the coverage of the 90% confidence intervals (90% CI coverage) and quantify how often invalid variance estimates are obtained (i.e. $\hat{\mathrm{Var}}({\hat{\beta }}_{1})<0$) for the Greenland/Steyerberg adaptation method. We simulated different degrees of available evidence and heterogeneity, and repeated each scenario 500 times. The corresponding results are presented in Table 2. An implementation in R of aforementioned methods is available on request.

**Table 2:** Results simulation study

				**No meta-analysis**			**Greenland/Steyerberg**				**Improved adaptation method**			**Improved adaptation method**		
							**adaptation method**			**(no prior)**				**(weakly informative prior)**		
***N*_I_**	***N*_L_**	***σ*_h_**	***ρ***(***x*_1_**, ***x*_2_**)	**PB**	**MSE**	**coverage**	**PB**	**MSE**	**coverage**	(*)	**PB**	**MSE**	**coverage**	**PB**	**MSE**	**coverage**
100	500	0	0	15.07%	0.613	89.0%	8.87%	0.219	89.2%	8	1.3 e+12%	1.8 e+23	97.8%	-1.98%	0.065	89.6%
200	500	0	0	6.58%	0.186	90.0%	2.34%	0.063	90.8%	1	18.13%	3.671	94.4%	-1.44%	0.043	89.0%
500	500	0	0	3.65%	0.061	90.4%	1.00%	0.024	90.0%	0	2.21%	0.026	91.0%	-0.54%	0.021	89.0%
1000	500	0	0	1.31%	0.028	90.2%	0.84%	0.014	91.2%	0	1.34%	0.014	90.6%	-0.11%	0.013	90.0%
100	500	0	0.50	20.39%	0.888	91.2%	5.75%	0.166	94.4%	7	-80.77%	3.9 e+04	98.4%	1.41%	0.048	96.2%
200	500	0	0.50	8.22%	0.226	91.0%	1.63%	0.037	93.0%	0	4.55%	0.091	94.2%	0.32%	0.031	93.6%
500	500	0	0.50	1.89%	0.073	87.6%	0.45%	0.019	92.2%	0	0.89%	0.020	90.8%	-0.32%	0.019	91.4%
1000	500	0	0.50	0.88%	0.031	92.2%	0.33%	0.011	93.8%	0	0.55%	0.012	92.8%	-0.19%	0.011	93.8%
100	500	0.20	0	10.89%	0.440	92.4%	5.17%	0.140	90.4%	8	-3.7 e+02%	5.6 e+04	98.0%	-4.02%	0.056	89.8%
200	500	0.20	0	6.54%	0.177	92.0%	3.81%	0.060	91.6%	1	-11.08%	0.801	95.6%	-0.18%	0.039	91.6%
500	500	0.20	0	1.23%	0.049	93.8%	0.34%	0.024	92.2%	0	1.53%	0.026	92.2%	-1.13%	0.022	90.8%
1000	500	0.20	0	0.94%	0.029	89.2%	0.89%	0.017	90.4%	0	1.42%	0.018	90.4%	0.02%	0.016	89.8%
100	2000	0	0	47.95%	4.9 e+01	93.2%	37.63%	4.3 e+01	86.2%	21	1.6 e+12%	1.5 e+23	98.2%	-1.09%	0.058	89.6%
200	2000	0	0	5.60%	0.184	90.2%	3.31%	0.058	89.8%	1	54.36%	2.1 e+02	94.2%	-0.12%	0.036	88.2%
500	2000	0	0	2.36%	0.064	87.2%	1.10%	0.017	89.2%	0	2.31%	0.020	91.4%	-0.07%	0.015	88.8%
1000	2000	0	0	1.17%	0.027	90.0%	0.58%	0.009	90.2%	0	1.16%	0.010	89.2%	-0.03%	0.009	87.4%
100	2000	0	0.50	20.05%	0.856	89.6%	5.68%	0.139	92.0%	11	3.5 e+12%	1.3 e+23	98.4%	1.67%	0.045	95.4%
200	2000	0	0.50	6.99%	0.206	90.8%	2.67%	0.035	92.2%	1	5.94%	0.120	93.8%	2.02%	0.029	92.2%
500	2000	0	0.50	2.44%	0.063	90.8%	0.75%	0.011	92.8%	0	1.18%	0.011	92.0%	0.45%	0.010	92.2%
1000	2000	0	0.50	1.62%	0.032	89.4%	0.26%	0.007	91.6%	0	0.45%	0.007	91.6%	0.02%	0.007	91.4%
100	2000	0.20	0	16.17%	0.654	92.6%	7.67%	0.201	89.8%	16	1.5 e+03%	3.9 e+04	98.2%	-2.66%	0.046	91.0%
200	2000	0.20	0	6.63%	0.177	93.0%	3.74%	0.057	89.2%	1	13.89%	0.754	94.8%	0.26%	0.037	88.8%
500	2000	0.20	0	2.33%	0.056	92.8%	1.23%	0.021	89.6%	0	2.46%	0.023	89.4%	-0.08%	0.019	88.6%
1000	2000	0.20	0	2.02%	0.027	92.2%	1.07%	0.014	87.4%	0	1.62%	0.015	86.6%	0.37%	0.013	85.8%

Simulation results for the situation in which an IPD of *N*_I_ subjects is available and the literature associations are based on 4 studies of *N*_L_ subjects each. Between-study heterogeneity of literature associations is parameterized by *σ*_h_. Correlation between the predictor variables *x*_1_ and *x*_2_ is indicated by *ρ*(*x*_1_, *x*_2_). The following statistics of ${\hat{\beta }}_{1}$ are presented: percentage bias (PB), Mean Squared Error (MSE) and coverage of the 90% confidence interval (coverage). We also assessed how often the Greenland/Steyerberg adaptation method estimated a negative variance for ${\hat{\beta }}_{1}$ (*).

### No meta-analysis (classical approach)

Results demonstrate that the classical approach to logistic regression, ignoring published univariable evidence from previous studies, considerably overestimates multivariable associations, particularly when the IPD at hand is very small. Although the percentage bias and MSE of ${\hat{\beta }}_{1}$ decreases in larger datasets, it does not completely disappear. Similar to previous research, we found that the bias of estimated regression coefficients increases when collinearity occurs and effective sample sizes are small [33]. The coverage of the 90% confidence interval was adequate for all scenarios considered.

### Greenland/Steyerberg adaptation method

The multivariable associations estimated with the Greenland/Steyerberg Adaptation method were far more accurate than those estimated with the classical approach, especially when little actual data were available. Estimated associations remain, however, too extreme compared to the associations from the reference model. The coverage of the 90% confidence interval was good for most scenarios, although we observed over-coverage when collinearity was present, and under-coverage when the literature studies were very large and heterogeneous. Unfortunately, we also noticed that some estimates for $\mathrm{Var}({\hat{\beta }}_{m|\text{L}})$ were negative when IPDs were small, and particularly when the literature studies were large (such that $\mathrm{Var}({\hat{\beta }}_{u|\text{L}})$ becomes negligible). Finally, the presence of heterogeneity in the literature associations did not influence the accuracy of estimated associations. This finding can however be explained by the fact that heterogeneity was only introduced in the spread of the literature associations.

### Improved adaptation method (no prior)

When no shrinkage was applied for the associations of the IPD at hand, estimated multivariable associations had the largest error, particularly when few data were available. Regression coefficients in bootstrap samples were often non-identifiable (results not shown), resulting in unstable estimates and over-coverage of multivariable regression coefficients. When the size of the IPD at hand increased, this approach performed similar to the improved adaptation method with a weakly informative default prior and the approach proposed by Greenland and Steyerberg.

### Improved adaptation method (weakly informative prior)

Results demonstrate that estimated associations were most accurate when a weakly informative prior was used during estimation of the adaptation. Even when the rule of thumb that logistic models should be used with a minimum of 10 outcome events per predictor variable is clearly violated, this approach yielded superior estimates of b_1_ that were very similar to estimates obtained from large amounts of IPD. Finally, we observed over-coverage of the 90% confidence interval when collinearity was present, and under-coverage when the literature studies were very large and heterogeneous with the IPD at hand.

## Application

We applied the methods discussed above to an empirical dataset of the prediction of peri-operative mortality (in-hospital or within 30 days) after elective abdominal aortic aneurysm surgery [22]. The study was exempted from ethical approval under Dutch law. Individual participant data were available for 238 subjects (including 18 deaths) and consisted of the predictors age, gender, cardiac co-morbidity (history of myocardial infarction, congestive heart failure, and ischemia on the ECG), pulmonary co-morbidity (COPD, emphysema or dyspnea) and renal co-morbidity (elevated preoperateive creatinine level). Univariable literature data were available from 15 studies with 15 821 subjects including 1 153 deaths in total (see Table, Additional file 1). We incorporated the univariable evidence from the literature data to estimate the multivariable associations of four of these predictors. Similar to the simulation study, we applied standard logistic regression modeling (no meta-analysis), the Greenland/Steyerberg Adaptation method and the improved adaptation method. The corresponding results are presented in Table 3.

**Table 3:** Calculation of adapted associations in the application

	**Female sex**	**MI**	**CHF**	**Ischemia**
**Adaptation**${\hat{\mu }}_{\delta }$**;**${\hat{\sigma }}_{\delta }^{2}$				
Greenland/Steyerberg Adapt. method	0.02; 0.13	-0.76; 0.07	-0.74; 0.05	-0.72; 0.08
Improved Adapt. method (no prior)	0.04; 0.39	-0.69; 0.15	-0.67; 0.16	-0.72; 0.41
Improved Adapt. method (weakly informative prior)	0.05; 0.12	-0.65; 0.07	-0.63, 0.05	-0.67; 0.11
**Univariable association**${\hat{\mu }}_{u}$**;**${\hat{\sigma }}_{u}^{2}$				
Greenland/Steyerberg Adapt. method	0.35; 0.03	1.02; 0.07	1.58; 0.12	1.52; 0.10
Improved Adapt. method (no prior)	0.35; 0.03	1.02; 0.07	1.58; 0.12	1.52; 0.10
Improved Adapt. method (weakly informative prior)	0.34; 0.03	1.00; 0.07	1.52; 0.11	1.48; 0.09
**Multivariable association**${\hat{\mu }}_{m}$**;**${\hat{\sigma }}_{m}^{2}$				
No meta-analysis	0.30; 0.75	0.74; 0.32	1.04; 0.35	0.99; 0.38
Greenland/Steyerberg Adapt. method	0.36; 0.16	0.26; 0.14	0.84; 0.17	0.80; 0.18
Improved Adapt. method (no prior)	0.38; 0.42	0.33; 0.22	0.91; 0.28	0.80; 0.51
Improved Adapt. method (weakly informative prior)	0.39; 0.15	0.35; 0.14	0.90; 0.16	0.81; 0.21

Illustration of the adaptation (Adapt.) methods for four independent associations for predicting peri-operative mortality (in-hospital or within 30 days) after elective abdominal aortic aneurysm surgery. The following estimates are presented: adaptation from univariable to multivariable association (with mean ${\hat{\mu }}_{\delta }$ and variance ${\hat{\sigma }}_{\delta }^{2}$), summary of univariable associations from the literature and IPD (with mean ${\hat{\mu }}_{\text{u}}$ and variance ${\hat{\sigma }}_{\text{u}}^{2}$) and adapted multivariable association (with mean ${\hat{\mu }}_{\text{m}}$ and variance ${\hat{\sigma }}_{\text{m}}^{2}$). Multivariable estimates were obtained through independent adaptation of the corresponding univariable associations, and are adjusted for the following variables: female sex, age in decades, history of myocardial infarction (MI), congestive heart failure (CHF), ischemia on electrocardiogram, renal co-morbidity and lung co-morbidity.

### No meta-analysis (classical approach)

The poor quality of estimated associations can be illustrated by their substantial variance. The predictor ‘Female Sex’ is a good example, since the 90% confidence interval of its multivariable association was estimated as [−1.30,2.00].

### Greenland/Steyerberg adaptation method

The Greenland/Steyerberg Adaptation method yielded notably different multivariable associations. For instance, whereas the classical approach estimated a multivariable association of 0.74 (OR_adj_ = 2.10) for the predictor ‘History of MI’, this estimate was shrunk to 0.26 (OR_adj_ = 1.20) by the adaptation method. Here, the considerable difference in univariable associations between the individual dataset and the literature is a major cause of shrinkage. Finally, the variance of multivariable associations was much smaller when published evidence from the literature was incorporated.

### Improved adaptation method (no prior)

We noticed a substantial increase in the variance of estimated adaptations due to the occurrence of non-identifiability in some of the bootstrap samples. These findings illustrate the need for a prior distribution that shrinks the associations of the individual dataset and thereby robustifies the adaptation.

### Improved adaptation method (weakly informative prior)

Multivariable associations were similar but not equal to those estimated with the Greenland/Steyerberg Adaptation method. For instance, the multivariable association of the predictor ‘History of MI’ was shrunk to a lesser extent by both variants of the improved adaptation method. Furthermore, the variance of estimated adaptations and multivariable associations decreased considerably by implementing a weakly informative prior distribution.

## Discussion

The incorporation of previously published univariable associations from single diagnostic or prognostic test, predictor or marker studies, into the development of a novel prediction model is both feasible and beneficial. A simple method for this purpose was proposed by Greenland and Steyerberg using the change from univariable to multivariable association observed in the IPD to adapt the univariable associations from the literature. We present an improved adaptation method and demonstrate its additional value in a simulation study. Particularly when the individual dataset is relatively small, this method estimates multivariable associations with a smaller MSE, and obtains better coverage of their 90% confidence intervals. Major performance gain is obtained by shrinking the associations from the individual dataset when calculating the adaptation. When no shrinkage was applied (no prior), non-identifiability occurred in some of the bootstrap samples and estimated adaptations were no longer normally distributed. Since we know that extreme associations are very rare in medical sciences, the use of a weakly informative default prior is justified [29], resulting in improved accuracy and precision of the adaptation and hence also the multivariable associations under study.

Several issues must be considered when evaluating these findings: Firstly, performance was evaluated here through the estimation of an association in a small prediction model. Our method may perform better in larger models where correlations between univariable and multivariable associations may be less strong, but this remains untested. Secondly, advanced Bayesian approaches for summarizing the evidence from the literature were not considered. Although these approaches might further improve the accuracy and coverage of multivariable associations, they are less readily compared with meta-analytical models and require more modeling expertise.

Third, the assumption that studies from the literature are exchangeable with the data at hand might not always hold. Simulations showed an under-coverage of the estimated 90% confidence interval when comparability between the considered associations was low, indicating that incorporating strongly heterogeneous evidence from the literature into prediction modeling remains problematic. In those scenarios, the change from univariable to multivariable association in the IPD at hand may no longer be representative for associations from the literature. Evidently, the incorporation of strongly heterogeneous evidence (for example indicated by the *I*^2^ statistic) from the literature into the development of a novel prediction model remains questionable [34,35]. In addition, aggregating published results may not be desirable if publication bias is present or suspected. Fortunately, the use of random effects when summarizing the associations from the literature seems to counter this problem to some extent.

Fourth, we did not consider the situation in which multivariable (rather than univariable) associations are available from the literature. Although their incorporation may be difficult due to the diversity of considered predictors, it could further improve the quality of estimated associations. The synthesis process of associations from the literature should then account for differences in model specification and included associations. Future research will investigate how these challenges can be assessed [36].

Finally, our simulation study only evaluated the performance of estimated multivariable predictor-outcome associations. Although Steyerberg *et al.* showed that improved estimates may increase the quality of the prediction model [19], this relation was not assessed here. It is possible that all adaptation methods perform similar in a prediction task. However, we showed that the Improved Adaptation Method with a weakly informative prior may further reduce the bias of multivariable associations when datasets are small. It may be clear that for strong predictors, this improvement may have a meaningful impact when making predictions. Additional research is needed to evaluate the extent to which improved predictor-outcome associations result in an improved model performance.

## Conclusions

Our study demonstrates that the MSE in multivariable associations of a novel prediction model is largest when external evidence, in this case previously published univariable predictor-outcome associations, is ignored. Although this error decreases with increasing amount of IPD, it does not disappear completely, even in very large datasets. Therefore, it is valuable to incorporate any existing univariable evidence from the literature unless this evidence is strongly heterogeneous. Even when the individual dataset is relatively large compared to the literature, the proposed method will still result in an estimate closer to the underlying multivariable association than the standard method ignoring the literature. The improved and original adaptation methods are robust approaches for this purpose. Whereas the latter method is simpler to apply, the former is more vigorous in small datasets and provides the most stable estimates.

## Competing interests

The authors declare that they have no competing interests.

## Author’s contributions

TD performed the statistical analyses and drafted the manuscript. DL contributed in the statistical models. HK and YV supervised the analyses and advised on several modeling issues. Finally, ES and KM provided critical feedback and streamlined the manuscript during the final stage. All authors read and approved the final manuscript.

## Funding

We gratefully acknowledge the financial support by the Netherlands Organization for Scientific Research (9120.8004 and 918.10.615 and 916.11.126).

## Pre-publication history

The pre-publication history for this paper can be accessed here:

http://www.biomedcentral.com/1471-2288/12/121/prepub

## Supplementary Material

Additional file 1 **Literature data from the application.** Reconstructed 2-by-2 tables of surgical mortality in relation to the preoperative characteristics gender, renal function, pulmonary function, history of MI, CHF and ischemia. Published studies and individual participant data (De Mol Van Otterloo) are shown, ordered by study size. Click here for file
