# Supplementary material for: Incorporating published univariable associations in diagnostic and prognostic modeling
Source: BMC Med Res Methodol. 2012 Aug 10;12:121. doi: 10.1186/1471-2288-12-121 (PMC3548751; doi:10.1186/1471-2288-12-121)
Supplement: Additional file 1 — Literature data from the application. Reconstructed 2-by-2 tables of surgical mortality in relation to the preoperative characteristics gender, renal function, pulmonary function, history of MI, CHF and ischemia. Published studies and individual participant data (De Mol Van Otterloo) are shown, ordered by study size. [file 1471-2288-12-121-S1.doc]

**APPENDIX 2**: Reconstructed 2-by-2 tables of surgical mortality in relation to the preoperative characteristics gender, renal function, pulmonary function, history of MI, CHF and ECG: Ischemia. Published studies and individual patient data (De Mol Van Otterloo) are shown, ordered by study size ^29^.

| **Gender**: | **Women** | | **Men** | |  | |
| --- | --- | --- | --- | --- | --- | --- |
| First author | Dead | Alive | Dead | Alive | $\beta_{u}$ | $\mathrm{var}(\beta_{u})$ |
| De Mol Van Otterloo | 2 | 19 | 15 | 202 | 0.279 | 0.620 |
| AbuRahma | 4 | 72 | 3 | 253 | 1.544 | 0.601 |
| Bosman | 1 | 40 | 29 | 291 | -1.383 | 1.063 |
| McCabe | 2 | 52 | 7 | 303 | 0.510 | 0.665 |
| Hannan | 66 | 712 | 207 | 2585 | 0.146 | 0.022 |
| Katz | 156 | 1313 | 457 | 6259 | 0.487 | 0.010 |

| **Renal function**: | **Impaired** | | **Unimpaired** | |  | |
| --- | --- | --- | --- | --- | --- | --- |
| First author | Dead | Alive | Dead | Alive | $\beta_{u}$ | $\mathrm{var}(\beta_{u})$ |
| Morishita | 1 | 41 | 2 | 66 | -0.217 | 1.540 |
| De Mol Van Otterloo | 3 | 12 | 15 | 208 | 1.243 | 0.488 |
| Bosman | 2 | 6 | 21 | 298 | 1.554 | 0.718 |
| McCabe | 3 | 42 | 6 | 313 | 1.315 | 0.527 |
| Diehl | 6 | 25 | 24 | 497 | 1.603 | 0.250 |
| Johnston | 22 | 201 | 10 | 340 | 1.314 | 0.153 |

| **Pulmonary function**: | **Impaired** | | **Unimpaired** | |  | |
| --- | --- | --- | --- | --- | --- | --- |
| First author | Dead | Alive | Dead | Alive | $\beta_{u}$ | $\mathrm{var}(\beta_{u})$ |
| Morishita | 3 | 33 | 0 | 74 | 1.937 | 1.378 |
| De Mol Van Otterloo | 6 | 39 | 12 | 181 | 0.842 | 0.281 |
| Diehl | 5 | 72 | 6 | 216 | 0.916 | 0.385 |
| Bosman | 8 | 63 | 20 | 267 | 0.528 | 0.195 |
| Johnston | 15 | 169 | 15 | 460 | 1.001 | 0.141 |

| **History of MI**: | **MI** | | **No MI** | |  | |
| --- | --- | --- | --- | --- | --- | --- |
| First author | Dead | Alive | Dead | Alive | $\beta_{u}$ | $\mathrm{var}(\beta_{u})$ |
| Fielding | 4 | 22 | 14 | 182 | 0.860 | 0.372 |
| De Mol Van Otterloo | 10 | 48 | 8 | 180 | 1.545 | 0.251 |
| Johnston | 13 | 147 | 19 | 487 | 0.818 | 0.138 |

| **Congestive Heart Failure**: | **CHF** | | **No CHF** | |  | |
| --- | --- | --- | --- | --- | --- | --- |
| First author | Dead | Alive | Dead | Alive | $\beta_{u}$ | $\mathrm{var}(\beta_{u})$ |
| De Mol Van Otterloo | 13 | 67 | 5 | 153 | 1.781 | 0.298 |
| Johnston | 8 | 46 | 24 | 588 | 1.449 | 0.190 |

| **Electrocardiogram**: | **Ischemia** | | **No Ischemia** | |  | |
| --- | --- | --- | --- | --- | --- | --- |
| First author | Dead | Alive | Dead | Alive | $\beta_{u}$ | $\mathrm{var}(\beta_{u})$ |
| De Mol Van Otterloo | 13 | 70 | 5 | 150 | 1.718 | 0.298 |
| Johnston | 12 | 80 | 20 | 554 | 1.424 | 0.148 |
